# Duck enteritis virus (DEV) UL54 protein, a novel partner, interacts with DEV UL24 protein

**DOI:** 10.1186/s12985-017-0830-5

**Published:** 2017-08-29

**Authors:** Xinghong Gao, Renyong Jia, Mingshu Wang, Qiao Yang, Shun Chen, Mafeng Liu, Zhongqiong Yin, Anchun Cheng

**Affiliations:** 1 Avian Disease Research Center, College of Veterinary Medicine, Sichuan Agricultural University, Chengdu, Sichuan 611130 China; 2 Key Laboratory of Infectious Disease & Bio-safety, Provincial Department of Education, Zunyi Medical University, Zunyi, Guizhou 563000 People’s Republic of China; 3 Institute of Preventive Veterinary Medicine, Sichuan Agricultural University, Chengdu, Sichuan 611130 China; 4 Key Laboratory of Animal Disease and Human Health of Sichuan Province, Chengdu, Sichuan 611130 China

**Keywords:** DEV, UL24 protein, UL54 protein, Protein-protein interaction (PPI)

## Abstract

**Background:**

UL24 is a multifunctional protein that is conserved among alphaherpesviruses and is believed to play an important role in viral infection and replication.

**Results:**

In this paper, to investigate putative UL24-binding proteins and to explore the functional mechanisms of DEV UL24, yeast two-hybrid (Y2H) was carried out, and further verified the interaction between UL24 and partners by co-immunoprecipitation and fluorescence microscopy experiments. Interaction partners of UL24 protein were screened by yeast two-hybrid (Y2H) with the cDNA library of DEV-CHv strain post-infection DEF cells. A novel partner, DEV UL54 protein, was discovered by Y2H screening and bioinformatic. Co-immunoprecipitation experiments suggested that DEV UL24 interacted with UL54 proteins. And distribution of a part of UL54 protein was changed from nucleus to cytoplasm in DF-1 cells of co-subcellular localization experiments which also showed that DEV UL24 interacted with UL54 proteins.

**Conclusions:**

The interaction between the DEV UL24 and UL54 proteins was discovered for the first time. Thus, DEV UL54 protein as a novel partner interacted with DEV UL24 protein.

**Electronic supplementary material:**

The online version of this article (doi:10.1186/s12985-017-0830-5) contains supplementary material, which is available to authorized users.

## Finding

Duck enteritis virus (DEV, anatid alphaherpesvirus 1 species), clustered in *mardivirus* genus, *alphaherpesvirinae* subfamily, *Herpesviridae* family according to the latest report of the International Committee on Taxonomy of Viruses (ICTV) [1], causes considerable economic losses to the commercial duck industry and poses a continuous threat to wild and migratory waterfowl populations (e.g., ducks, geese and swans) due to their high mortality and decreased egg production rates [2].

Currently, there are three complete genomic sequences of DEV strains available in GenBank: the Chinese virulent DEV strain (DEV CHv) [3, 4], the European virulent strain (2085) [5], and the attenuated vaccine strain (VAC) [6], and the publications related to the three genome sequences have cast light on the genome structure of DEV. DEV is a linear, double-stranded DNA virus, the genome size of which is approximately 158–162 kb [3–6]. The entire genome of DEV is composed of a unique long (UL), a unique short (US) and two inverted repeated sequences (IRS and TRS) [6]. A total of 78 ORFs were predicted to code for the potential functional proteins. Of these ORFs, 10 and 68 ORFs coded for structural proteins and non-structural proteins, respectively. Many DEV proteins, such as UL16 [7], UL38 [8], gE [9], gN [10, 11] have been researched in molecular biology studies. However, these researches on protein-protein interaction (PPI) were only done between gM and gN [10]. And there was no report on partners of DEV UL24 protein.

UL24 protein is a conserved multifunctional protein and is believed to play an important role in viral infection and replication. UL24 protein contains five homology domains (HDs) with a high percentage of amino acid identity among its homologs of the other Herpesvirus family members (including HSV1/2 UL24, EHV-1 ORF37, HCMV UL76, MHV-68 ORF20, and so on) and one PD-(D/E)XK endonuclease motif in the N-terminal regions (NTRs) [12–14]. Using mouse infection model, researches showed that HSV-1 UL24 protein was involved in viral pathogenesis [15, 16] and contributed to viral replication in the mucous membranes [17]. ORF 37 is a neuropathogenic determinant of equine herpesvirus 1 (EHV-1) [18, 19]. UL76 protein of human cytomegalovirus (HCMV) was able to induce DNA double-strands breaks and DNA damage response [20–22]. ORF 20 of murine herpesvirus 68 (MHV-68) was reported to be involved in inducing cell-cycle arrest at the G2/M phase followed by apoptosis [13, 23]. In summary, UL24 protein contributes to virus virulence [16, 17, 19], viral replication [15, 24–26], cell membrane fusion [27, 28], cell cycle arrest [23], and redistribution of nucleolin (C23) and nucleophosmin (B23) [27–31]. Up to now, research on DEV UL24 protein showed that it is located in the cytoplasm around the periphery of the nucleus in DEV-infected DEF cells [32]. And attenuated Salmonella *Typhimurium* delivering DNA vaccine encoding DEV UL24 induced immune responses and conferred good protection against challenge [33, 34].

The DEV UL54 is an immediate early gene [33], but its function is not very clear. Bioinformation analysis showed that DEV UL54 encode a 51.75 KDa protein of 458 AA with 56% homology to the corresponding HSV-1 protein ICP27. ICP27, a conserved and multifunctional protein, is characterized nucleocytoplasmic shuttling based on crucial nuclear localization signal (NLS) and nuclear export signal (NES) [35–37]. ICP27 has been implicated in viral replication [35, 38], gene expression [39, 40], apoptosis [41] and host immunization reactions [42, 43], all of which promote infection. Thus, these features of ICP27 provide ideas or inspiration for research on UL54.

UL24 protein family is a multifunctional protein playing important roles in herpesvirus invasion and replication. However, there are only a few reports on the molecular mechanisms underlying the function of UL24 protein [32, 34, 44, 45]. Thus, study on PPI of DEV UL24 contributes to better understanding of functions and molecular mechanisms of this protein, which also prompts us to understand the molecular mechanisms of DEV infection. To this end, we employed yeast two-hybrid technology coupled with co-immunoprecipitation to screen DEV UL24 protein interacting partner.

**Fig. 1:**
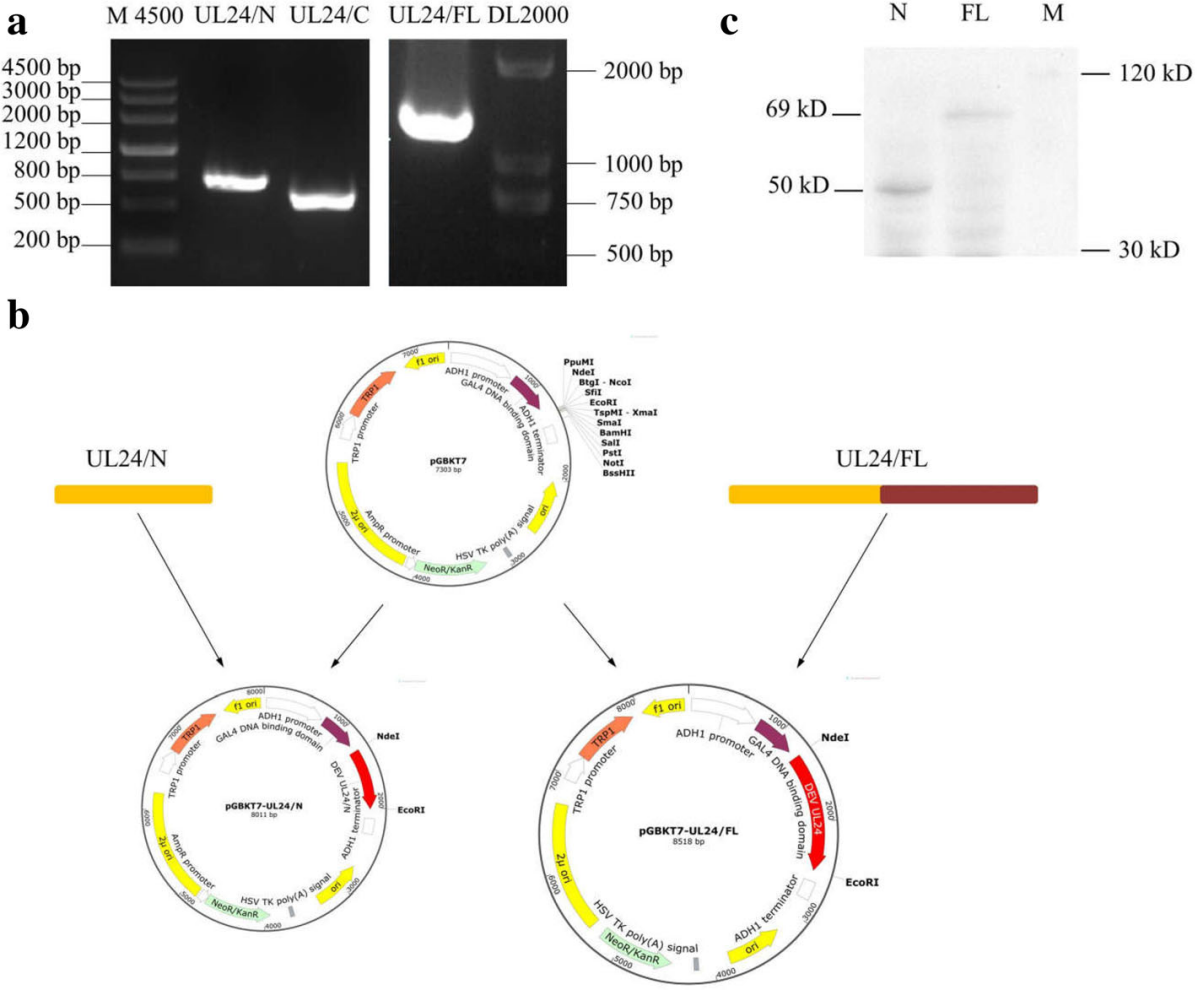
Bait plasmid construction. **a**, the nucleotide sequence of UL24/C (721 ~ 1230 bp, lane 3) and codon optimized UL24/N (1 ~ 720 bp, lane 2) was firstly amplified by PCR. Then, the full length codon optimized UL24 gene (1 ~ 1230 bp, lane 4) was amplified with the DNA fragments UL24/C and UL24/N as template by overlap PCR. M4500: DNA Marker 200 ~ 4500 bp. DL2000: DNA Marker 100 ~ 2000 bp. **b**, “bait” proteins (N and FL) were expressed in *Saccharomyces cerevisiae* Y2HGold strain according to be detected by western blotting. Primary antibody (mouse-anti myc mAb) is diluted 1000 folds, and HRP-goat anti-mouse antibody is diluted 80,000 folds. Immunoreactive proteins are detected using the ECL kit (enhanced chemiluminescence system, Bio-Rad)

Sequence analysis of the N-terminal region of DEV UL24 gene (nucleotides 1–720, Additional file 1: Figure S1) was carried out by codon optimization with host yeast of *Saccharomyces cerevisiae* (http://www.jcat.de/). Optimized sequence was generated by company of Huada (China). To clone full-length optimized DEV UL24 gene, two pairs of primer were designed (Table 1, primers UL24/N-F/R, UL24/C-F/R). Viral sequences (N-terminal fragment, UL24/N; and full-length optimized DEV UL24 gene, UL24/FL) were cloned into pGBKT7 plasmid (bait plasmid; Clontech) and Y2HGold strain (bait strains; Clontech) was transformed with this two recombinant plasmids (Fig. 1a, b), respectively. UL24/N strain was used as a control. Then, bait strains were verified for self-activation, toxicity and Western blot analysis according to protocols as described in Matchmaker Gold Yeast Two-Hybrid System User Manual (Clontech) [46, 47].Self-activation and toxicity detection of bait strains were negative. Western blot analysis revealed that UL24/N and UL24/FL-fusion proteins were expressed. Based on theoretical estimates, UL24/N and UL24/FL-fusion proteins (contain GAL4 DNA binding domain of pGBKT7 plasmid about 22 kDa; myc flag protein about 1 kDa) are about 50 kDa, 69 kDa respectively (Fig. 1c).

**Table 1:** primer sequences

primer name	sequence	restriction enzyme
UL24/N-F	5′-AGGAGGACCTG**CATATG**ATGGCTTCTAAGGTTCAAAAGAAGAGA-3’	Nde I
UL24/N-R	5′-GGATCCCCGG**GAATTC**TGGTATTCAGACAAACCAG-3’	EcoR I
UL24/C-F	5′-ATCGCTGGTTTGTCTGAATACCACATACCTACCAAAGGTAAGCGCCGG-3’	——
UL24/C-R	5′-GGATCCCCGG**GAATTC**CTAGTGTTTAGTTGGTCTGA	EcoR I
pCMV-myc-UL24 F	5′-ATGGAGGCCC**GAATTC**GGATGGCATCGAAGGTACAGA-3’	EcoR I
pCMV-myc-UL24 R	5′-GCCGCGGTAC**CTCGAG**ACTAGTGTTTAGTTGGTCTGAA-3’	Xho I
pCMV-Flag-UL54 F	5′-**CATATG**ATGGCCTGCAGTGCTAAA-3’	Nde I
pCMV-Flag-UL54 R	5′-**GGATCC**CAAACATTTCATTACAATAAAA-3’	BamH I
pEGFP-N1-UL24-F1	5′-AAGCTTC**GAATTC**TGATGGCATCGAAGGTACAGA-3’	EcoR I
pEGFP-N1-UL24-R2	5′-CGACCGGTGGAT**CCCGGG**CGTGTTTAGTTGGTCTGAATA-3’	Sma I
pDsRed-N1-UL54-F1	5′- TCTC**AAGCTT**AAGCTATGGCCTGCAGTGCTAAAC-3’	Hind III
pDsRed-N1-UL54 R2	5′- GGCGACCGGT**GAGCTC**GTAAACATTTCATTACAATA-3’	BamH I

The restriction enzyme sites were bold

**Fig. 2:**
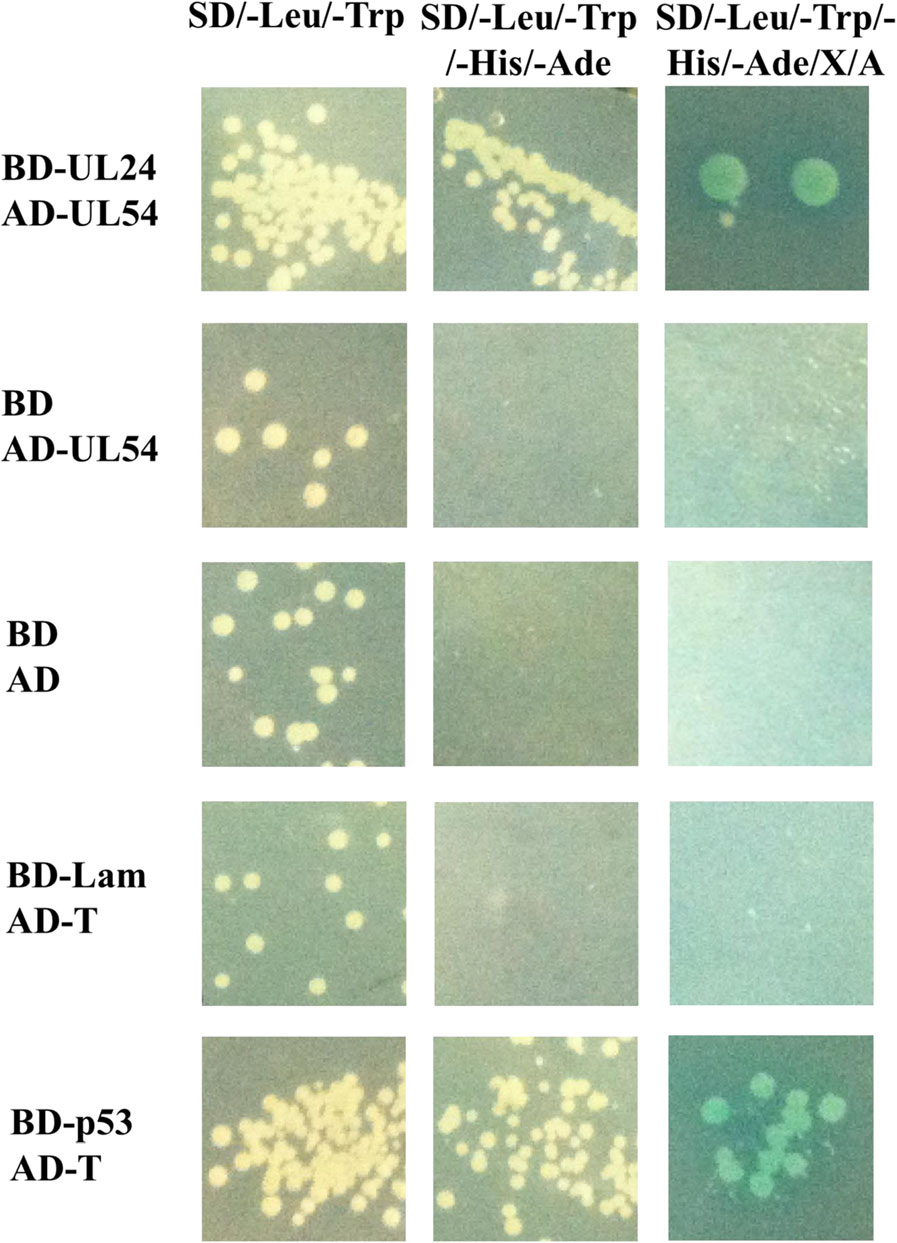
Mapping of the UL24/UL54 interaction. Candidate UL54 protein was identified by Y2H assay. Empty bait vector (pGBKT7) and prey vector pGADT7-UL54 were used to self-activation detection of UL54 protein. Bait vector pGBKT7 and prey vector pGADT7 were used as a blank control. Bait vector pGBKT7-Lam and prey vector pGADT7-T were used as a negative control. Bait vector pGBKT7-p53 and prey vector pGADT7-T were used as a positive control. A positive interaction is indicated by the production of a blue yeast colony in the SD/−Leu/−Trp/−His/−Ade/X/A plates

To explore the functional mechanisms of DEV UL24 and to investigate putative UL24-binding proteins, Y2H screens were performed by mating (according to protocol in Matchmaker Gold Yeast Two-Hybrid System User Manual, Clontech). Briefly, the cDNA library was constructed by previously described, which was comprised all genes of the DEF cells post-infection DEV-CHv strain and contained more than 10^7^ primary clones per milliliter [48]. Four putative interacting proteins, DEV UL54 (Accession: EU071033.1), duck PSF2 (Accession: XM_013096619.1), GNB2L1 (Accession: XM_005018317.2), and *Anas platyrhynchos* Nudix-type motif 9 (Accession: XM_005012818.2) were obtained by sequencing analysis and NCBI (National Center for Biotechnology Information) blast analysis. NCBI blast analysis suggested that the first base of positive clone contained GNB2L1 mRNA sequence, and matched with the 426th base of GNB2L1 mRNA sequence; the 78th base of positive clone contained *Anas platyrhynchos* Nudix-type motif 9 mRNA sequence and matched with the 194th base of *Anas platyrhynchos* Nudix-type motif 9 mRNA sequence. According to triplet code characteristic of nucleic acid code protein, we concluded the positive clones which contained GNB2L1 mRNA sequence and *Anas platyrhynchos* Nudix-type motif 9 sequences as probably frame-shift mutants. PSF2 sequence lay in 3’UTR of duck PSF2 mRNA whereas DEV UL54 sequence unaffected. Thus, we used positive clone which contains UL54 sequence to eliminate the false positive of it by Y2H (Fig. 2). DEV UL54 mRNA also contained a polyA site that was 26 nt downstream of the UL54 CDS region.

DEV UL24 and UL54 sequences were cloned by recombination into pCMV-myc-N and pCMV-Flag-N vectors respectively. Primers were designed and are listed in Table 1 (primers pCMV-myc-UL24 F/R, pCMV-Flag-UL54 F/R). Positive clones were identified by sequencing. HEK293T cells (Human embryonic kidney cells, HEK) were cultured in DMEM (Dulbecco modified Eagle medium, Gibco) supplemented with 10% (*v*/v) FBS (fetal bovine serum, Gibco), 100 units/mL penicillin, and 100 μg/mL streptomycin in an atmosphere of 5% CO_2_ at 37 °C. For co-expression DEV UL24 and UL54 proteins, equal plasmids were transiently co-transfected into HEK293T cells using a Lipofectamine-2000 transfection reagent system (Invitrogen). Total protein was harvested at 48 h post-transfection by incubating cells for 30 min on ice, followed by scraping into NP-40 lysis buffer with the addition of ionic detergents (0.5% sodium deoxycholate and 0.1% SDS) [35]. Debris was pelleted by centrifugation at 13,000×g for 20 min in 4 °C. Western blot (WB) analysis of cell extracts revealed that both UL24 and UL54 fusion proteins were expressed with molecular weights of about 45 kDa and 50.5 kDa, respectively (Additional file 2: Figure S2). The full length UL24-fusion protein was expressed and about 69 kDa (containing 22 kDa binding domain of GAL4 protein and 1 kDa myc tagged protein) in *Saccharomyces cerevisiae* Y2HGold strain firstly according to codon optimization (Additional file 1: Figure S1). Thus, full length DEV UL24 protein was about 46 kDa in Y2HGold strain which was consistent with expected results. Therefore, DEV UL24 protein expressed in eukaryote is about 45 kDa.

**Fig. 3:**
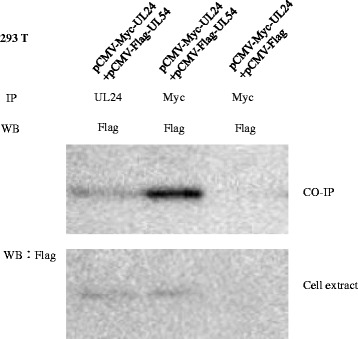
Co-immunoprecipitation of UL24 and UL54. HEK293T cells were co-transfected with eukaryotic plasmids (pCMV-myc-UL24 and pCMV-Flag-UL54, pCMV-myc-UL24 and pCMV-Flag respectively). At 48 h post-transfection, rabbit anti-UL24 pAb were incubated with the extracts from the cells co-transfected with the plasmids pCMV-myc-UL24 and pCMV-Flag-UL54; Abs against myc (mouse against myc) were incubated with extracts from the cells co-transfected with pCMV-myc-UL24 and pCMV-Flag-UL54, pCMV-myc-UL24 and pCMV-Flag-N respectively. Immunoprecipitated complexes were analyzed by western blotting with mouse anti-Flag pAb. The above transfected 293 T cell extracts were analyzed by western blotting with mouse anti-Flag pAb to detect the expression of Flag-UL54

Immunoprecipitation was performed with 2 mg of total protein incubated with 5 μL myc-agarose (mouse-anti-myc monoclonal antibody coupling with agarose, Santa Cruz Biotechnology) for 2 h at 4 °C, or incubated with 3 μg rabbit anti-UL24 antibody (polyclonal antibody, pAb) for 2 h at 4 °C. And compounds which contained pAb UL24 were incubated with protein A&G plus agarose (Santa Cruz Biotechnology) for another 2 h at 4 °C. Then, the other steps of immunoprecipitation were performed as protocol [49]. According to WB analysis, we observed that UL54 fusion protein, in above transfected 293 T cell extracts, was expressed in the experimental group (Fig. 3, lane 1&2) in contrast to control where no visible band was detected (Fig. 3, lane 3). Simultaneously, cell extracts were precipitated using rabbit-anti DEV UL24 antibody coupled to protein A &G-agarose and precipitated with myc-agarose respectively. Subsequent WB analysis showed that UL54 fusion protein was detected in the experimental group (Fig. 3, lane 1&2), but not in the control group (Fig. 3, lane 3). Furthermore, the intensity of the UL54 fusion protein in lane 2 was greater than in lane 1. To summarize, using co-immunoprecipitation, our results suggested that UL24 and U54 proteins interact with each other.

**Fig. 4:**
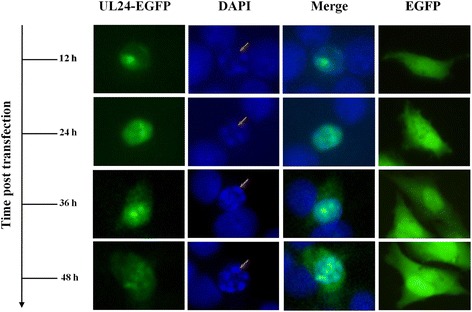
Subcellular localization of UL24 protein in DF-1 cells. DF-1 cells were transfected with pEGFP-N1-UL24. 12 h, 24 h, 36 h and 48 h after transfection, cells were fixed, permeabilized and then stained with 4′,6-diamidino-2-phenylindole (DAPI). The subcellular localization of UL24 protein was visualized using fluorescent microscopy. The DF-1 cells were transfected with the empty vector pEGFP-N1 as a negative control

**Fig. 5:**
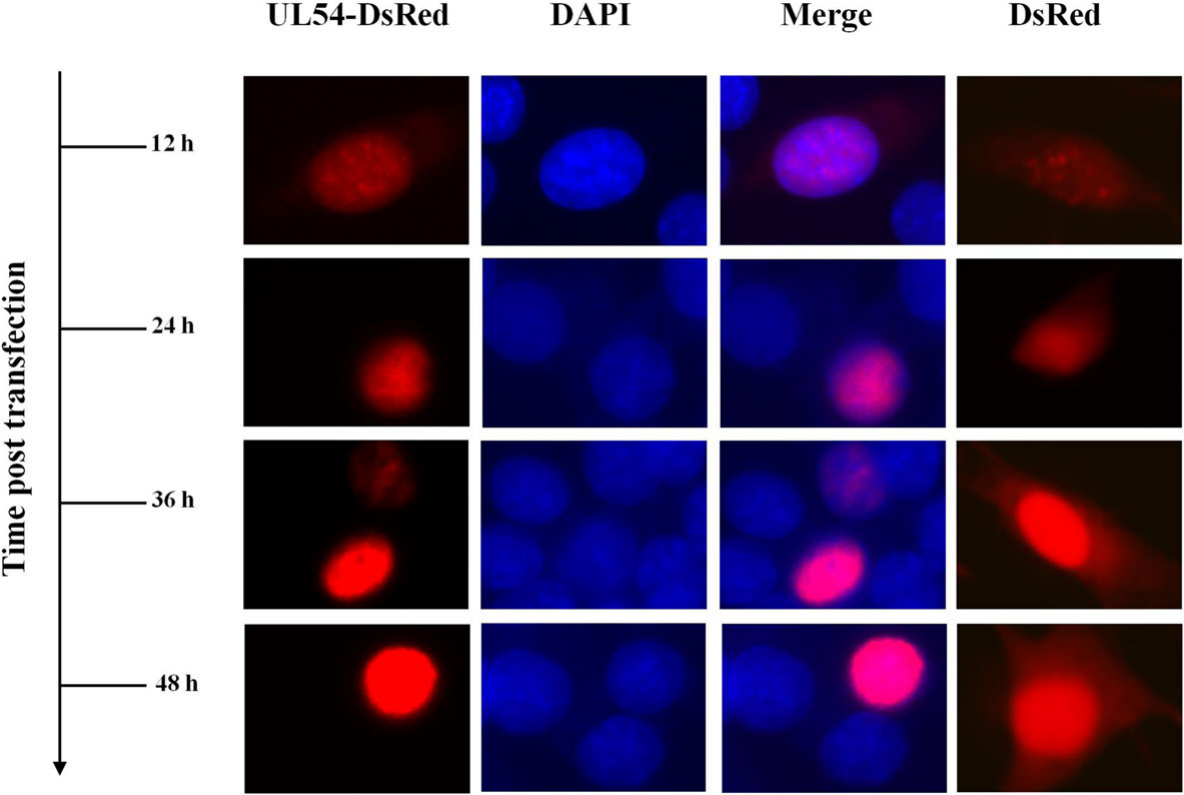
Subcellular localization of UL54 protein in DF-1 cells. After infection pDsRed-N1-UL54 plasmids 12 h, 24 h, 36 h and 48 h respectively, DF-1 were fixed, hyalinized and DAPI stained. And fluorescence microscopy was visualized directly. Meanwhile, DF-1 cells were transfected with pDsRed-N1 as a negative control

To further verify the interaction between UL24 and UL54 protein and to explore the function of UL24 protein during DEV infection, fluorescence microscopy was carried out. DEV UL24 and UL54 sequences were cloned by recombination into pEGFP-N1 and pDsRed-N1 vectors respectively. Primers were designed as in Table 1 (primers pEGFP-N1-UL24 F/R, pDsRed-N1-UL54 F/R). Positive clones were identified by sequencing. Chicken fibroblast cells (DF-1) were cultured and transiently transfected/ co-transfected as same as HEK293T cells. Respectively, 12 h, 24 h, 36 h, 48 h after transfection, the transfected cells were fixed in 4% paraformaldehyde for 10 min, permeabilized treatment in 0.3% Triton X-100 for 10 min, stained in 10 μg/mL DAPI solution (Sigma) for 8 min, and observed with a fluorescence microscope under a × 40 objective [50]. Results in Fig. 4 also demonstrated that UL24-EGFP protein was localized predominantly to the nucleus but a fraction also appeared to be located in the cytoplasm at 36 h and 48 h, post-transfection. Simultaneously, significant nuclear fragmentation was observed at 12 h48 h post-transfection as revealed by DAPI stain. Thirdly, UL24-EGFP protein distribution exhibited a globular shape or crystal shape aggregation. Figure 5 showed that UL54-DsRed protein was located in nucleus predominantly.

**Fig. 6:**
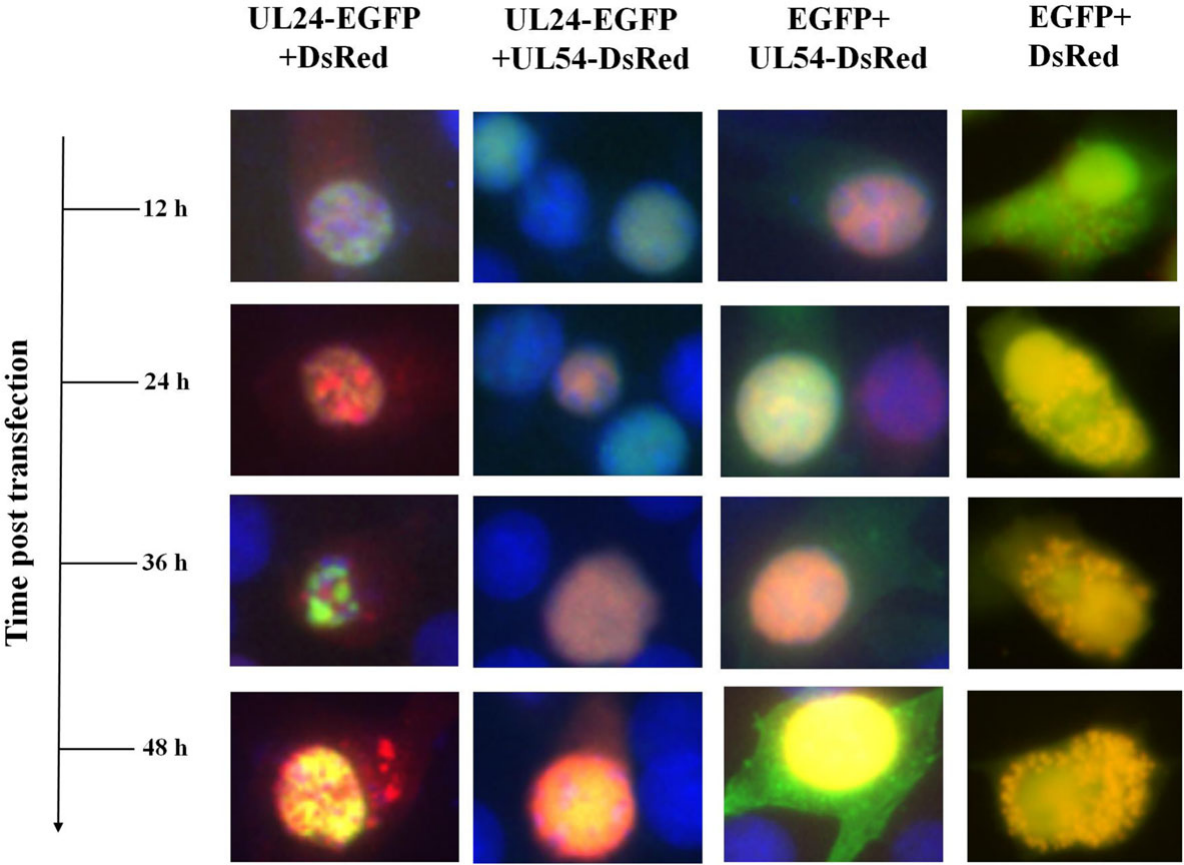
Subcellular co-localization of UL24 and UL54 protein in DF-1 cells. DF-1 cells were co-transfected with equal pEGFP-N1-UL24 and pDsRed-N1, pEGFP-N1 and pDsRed-N1-UL54, pEGFP-N1-UL24 and pDsRed-N-UL54, pEGFP-N1 and pDsRed-N1 respectively. 12 h, 24 h, 36 h and 48 h after infection, cells were fixed, hyalinized and DAPI stained. Fluorescence microscopy was visualized directly

Subcellular co-localization of UL24- and UL54-fusion proteins, at 12 h ~ 36 h post-transfection, UL24-EGFP and UL54-DsRed proteins was redistributed equably in nucleus. At 48 h post-infection, UL54-DsRed proteins were partly transported to cytoplasm although most of UL24- and UL54-fusion proteins were distributed equably in nucleus (Fig. 6, UL24-EGFP + UL54-DsRed group). Furthermore, some cell nucleus, in which UL24-EGFP and UL54-DsRed proteins were redistributed equably, was not a form of visible micronucleus by fluorescence microscopy. For in groups of negative control (UL24-EGFP+ DsRed group, EGFP + UL54-DsRed group), UL24-EGFP and UL54-DsRed proteins were predominantly laid in nucleus, respectively (Fig. 6, list 1&3). The blank control group (EGFP + DsRed group) revealed that EGFP and DsRed co-located in cytoplasm and nucleus equably (Fig. 6, list 4). In summary, our data suggested that UL24- and UL54-fusion protein could be co-expressed in DF-1 cells, and that the redistribution of UL54-fusion protein was caused by interactions between UL24 and UL54 protein.

In the past years, it has been reported that the HSV-1 ICP27 protein, a homologue of DEV UL54 protein [38, 51–53], can shuttle from the nucleus to the cytoplasm due to interaction with the host Nup62 protein [35]. The interactions between ICP27 protein and Nup62 protein inhibits host mRNAs exported to cytoplasm and regulates the expression of virus genes via regulation of the amount of the virus mRNAs exported to cytoplasm [35, 54]. HSV-1 UL24 protein is located in the nucleus, nucleolus and cytoplasm [55], whereas DEV UL24 protein is localized in the cytoplasm and the nucleus [34, 44]. UL24 protein interaction with UL54 protein existed in other five herpesviruses [56]. Therefore, we infer that the function of UL24 and UL54 PPI in DEV was probably the same as UL24 and ICP27 in HSV-1.

The UL24 protein is located differently in different cells. In this article, the localization of UL24-EGFP fusion protein was in the nucleus and cytoplasm in DF-1 cells (Fig. 4). In previous study, DEV UL24-LTB fusion protein was also located in the nucleus and cytoplasm when it was transiently expressed in COS-7 cells [34]. When overexpressed in COS-7 or DF-1 cells, UL24-fusion protein was predominantly localized in the nucleus. But DEV UL24 protein was located in the perinuclear region in DEV infected DEF cells, and regardless of an earlier or later time-point in infection, a little UL24 protein was observed in the nucleus [32]. It was guessed that there was a protein which interacted with DEV UL24, and UL24 could made it shuttle from nucleus to cytoplasm during DEV infection. DEV UL54 protein had a characterization of nucleocytoplasmic shuttling [57], and DEV UL24 interacted with UL54. Thus, we concluded that DEV UL54 probably promoted UL24 transportation from nucleus to cytoplasm during DEV infection.

Overexpression of UL24-fusion protein in DF-1 cell, induced DNA fragmentation and formation micronucleus according to DAPI stain (Fig. 5 and Additional file 2: Figure S2), suggested DEV UL24 protein functions in DNA damage. Similarly, HCMV UL76 protein, a homologue of DEV UL24 protein in *herpesvirus* family could induce DNA fragmentation and a form of micronucleus [21, 22]. Co-subcellular localization of UL24 and UL54-fusion protein, and redistribution of UL24-EGFP and UL54-DsRed proteins were changed at time post-transfection. Thus, we concluded that the redistribution of UL24 and UL54-fusion protein was caused by interactions between UL24 and UL54 protein. Interestingly, in cells of co-expression UL24 and UL54 proteins, some nucleus did not have DNA damage (Fig. 6, list 2). It suggested that the PPI between UL24 and UL54 protein could reduce the effect of DNA damage, and we would explore the molecular mechanism in the future.

## Conclusions

UL24 is a multifunctional protein, playing important roles in virus invasion and replication. To identify the molecular mechanisms underlying the function of UL24 protein, Y2H experiment coupled with CO-IP and co-subcellular localization were employed. UL54 protein, as a novel partner, interacted with DEV UL24 protein and conserved in *herpesviridae* family. In addition, the redistribution of partial UL54 proteins took changes from nucleus to cytoplasm, and the micronucleus disappeared in some of co-expression DF-1 cells. We concluded that the interaction between the two proteins is associated with several pathogenic processes in DEV infection, such as DNA damage and viral replication. And the molecular mechanism of this interaction contribution to DEV pathogenic infection is required to be further researched in the future.

## Additional files

Additional file 1: Figure S1. UL24/N gene codon optimization (1 ~ 720 bp). The green short-line was optimized nucleotides. (PDF 312 kb)

Additional file 2: Figure S2. WB analyzed the expression of UL24-fusion protein and UL54-fusion protein in HEK293T cells. HEK293T cells were transfected with eukaryotic plasmid pCMV-myc, pCMV-myc-UL24, and pCMV-Flag-UL54 respectively. At 48 h post-infection, the 293 T cell extracts were carried out Western blotting analysis, which indicated that myc-UL24 and Flag-UL54 was expressed in 293 T cells and the molecular mass of fusion protein is about 45 KD, 50.5 KD respectively. Primary Abs against myc-UL24 and Flag-UL54 were serums of rabbit against UL24 and mouse against Flag respectively. (PDF 44 kb)

## Abbreviations

CO-IP: Co-immunoprecipitation
CPE: Cytopathic effect
DEV: Duck enteritis virus
DMEM: Dulbecco modified Eagle medium
EHV-1: Equine herpesvirus 1
FBS: Fetal bovine serum
HCMV: Human Cytomegalovirus
HDs: Homology domains
HEK cells: Human embryonic kidney cells
HSV-1: Herpes simplex virus 1
ICP27: Infectious cell protein 27
ICTV: International Committee on Taxonomy of Viruses
IRS: Inverted repeated sequences
MHV-68: Murine Herpes Virus 68
NBCS: Newborn calf serum
NES: Nuclear export signal
NLS: Nuclear localization signal
NTRs: N-terminal regions
ORF: Open reading frame
PCR: Polymerase chain reaction
PPI: protein-protein interaction
UL: Unique long region
US: Unique short
Y2H: Yeast two-hybrid
