# Supplementary material for: Duck enteritis virus (DEV) UL54 protein, a novel partner, interacts with DEV UL24 protein
Source: Virol J. 2017 Aug 29;14:166. doi: 10.1186/s12985-017-0830-5 (PMC5575879; doi:10.1186/s12985-017-0830-5)
Supplement: Supplementary file 1 — UL24/N gene codon optimization (1 ~ 720 bp). The green short-line was optimized nucleotides. (PDF 312 kb) [file 12985_2017_830_MOESM1_ESM.pdf]

Alignment Report of Untitled ClustalW (Slow/Accurate, IUB)

Tuesday, October 13, 2015 10:57 AM

```
Majority               ATGGCTTCTAAGGTTCAGAAGAAGCGCGCTGGTTTGTGTGTTCGCAAGTGTCGCGCTGTTACTTTTGATCGGAAGTGTAT
                                10        20        30        40        50        60        70        80
DEV UL24-MMYH(N).seq   ATGGCTTCTAAGGTTCAAAAGAAGAGAGCTGGTTTGTCTGTTAGAAAGTCTAGAGCTGTTACTTTCGACAGAAAGTGTAT 80
DEV UL24(N).seq        ATGGCATCGAAGGTACAGAAAAAGCGCGCCGGATTAAGCGTTCGCAAGAGTCGCGCAGTGACATTTGATCGGAAATGTAT 80

Majority               TGGTGCTGCTACTACTGCTGCTTTTAAGAACGCTGAACAGCGGCCTTGTAGACGCGTTGCTGGTTCTAAGAGACGAACTG
                                90       100       110       120       130       140       150       160
DEV UL24-MMYH(N).seq   CGGTGCTGCTACTACTGCTGCTTTGAAGAACGCTGAACAAAGACCATGTAGAAGAGTTGCTGGTTCTAAGAGAAGAACTG 160
DEV UL24(N).seq        TGGCGCAGCGACGACAGCCGCACTTAAGAACGCGGAACAGCGGCCTTGTAGACGCGTAGCAGGATCCAAGAGACGAACCG 160

Majority               ATGCTAACACTTCTGGTTCTGTTGCTCGTCGCTTGAAGGCTGGTGTTCGCTGTCACAATCGTTTTTATGGTGCTTTGGCT
                               170       180       190       200       210       220       230       240
DEV UL24-MMYH(N).seq   ACGCTAACACTTCTGGTTCTGTTGCTAGAAGATTGAAGGCTGGTGTTAGATGTCACAACAGATTCTACGGTGCTTTGGCT 240
DEV UL24(N).seq        ATGCCAACACGTCCGGTTCAGTCGCACGTCGCCTCAAAGCTGGAGTGCGCTGCCACAATCGTTTTTATGGCGCCCTGGCT 240

Majority               AACGATTTGAACAAGTTTCGTTCTAATGGTGGTTCTCCCTGTAAGTTGATTGTTCGGTTGTTCGAACACACTTTGTCTTT
                               250       260       270       280       290       300       310       320
DEV UL24-MMYH(N).seq   AACGACTTGAACAAGTTCAGATCTAACGGTGGTTCTCCATCTAAGTTGATCGTTAGATTGTTCGAACACACTTTGTCTTT 320
DEV UL24(N).seq        AACGATTTGAACAAATTTCGTTCAAATGGTGGATCGCCCAGTAAACTGATTGTGCGGCTATTCGAACACACTCTGTCTTT 320

Majority               GCAGACTTTTAAGACTGCTTTTGATGTTAGGTTGACTTTTGAAGTTAACTTGGGTAGGCGCCGCCCAGATTGTATCTGTA
                               330       340       350       360       370       380       390       400
DEV UL24-MMYH(N).seq   GCAAACTTTGAAGACTGCTTTCGACGTTAGATTGACTTTCGAAGTTAACTTGGGTAGAAGAAGACCAGACTGTATCTGTA 400
DEV UL24(N).seq        GCAGACACTTAAAACTGCATTTGATGTGAGGCTGACTTTTGAAGTCAACTTAGGAAGGCGCCGCCCAGATTGCATATGCA 400

Majority               TGATTAAGACTGGTGAGGCTGAAACTGCTGATGTTATCTGTATTATTTTGGAATTGAAGACTTGTAAGTTTGTTAAGAAT
                               410       420       430       440       450       460       470       480
DEV UL24-MMYH(N).seq   TGATCAAGACTGGTGAAGCTGAAACTGCTGACGTTATCTGTATCATCTTGGAATTGAAGACTTGTAAGTTCGTTAAGAAC 480
DEV UL24(N).seq        TGATTAAGACAGGCGAGGCAGAAACTGCAGATGTCATATGTATTATTTTGGAACTCAAGACTTGCAAGTTTGTAAAAAAT 480

Majority               ATGACTACTGGTTGTAAGCAACAGCAAACTTGGACTGGTATTAGACAATTGATGGAATCTGCTATCTTGTTGGAACGTAT
                               490       500       510       520       530       540       550       560
DEV UL24-MMYH(N).seq   ATGACTACTGGTTCTAAGCAACAACAAACTTGGACTGGTATCAGACAATTGATGGAATCTGCTATCTTGTTGGAAAGAAT 560
DEV UL24(N).seq        ATGACAACAGGAAGTAAACAACAGCAAACATGGACTGGAATTAGACAATTGATGGAATCGGCCATCTTGTTGGAACGTAT 560

Majority               CATGCCCCCTGGTTGTTGTCAATTTTTGATCTGTCCGTTGTTGGTTTTTGTTGCTCAGCGCGGTTTGGACATCTTGCGAA
                               570       580       590       600       610       620       630       640
DEV UL24-MMYH(N).seq   CATGCCACCAGGTTGTTCTCAATTCTTGATCTGTCCATTGTTGGTTTTCGTTGCTCAAAGAGGTTTGGACATCTTGAGAA 640
DEV UL24(N).seq        CATGCCCCCTGGATGCAGTCAATTTTTAATATGTCCGTTGCTAGTATTTGTAGCCCAGCGCGGTCTGGACATACTCCGAA 640

Majority               TTACTCGATTTGCTCATCGGCGCGTTTCTTCTAACTTTGCTGCTTTGTCTGCTTCTATTGCTGGTTTGTCTGAATATCAT
                               650       660       670       680       690       700       710       720
DEV UL24-MMYH(N).seq   TCACTAGATTCGCTCACAGAAGAGTTTCTTCTAACTTCGCTGCTTTGTCTGCTTCTATCGCTGGTTTGTCTGAATACCAC 720
DEV UL24(N).seq        TTACACGATTTGCTCATCGGCGCGTATCCTCCAACTTTGCGGCATTATCAGCCTCTATTGCAGGTTTATCAGAATATCAT 720
```
