# Supplementary figures and images for: Duck enteritis virus (DEV) UL54 protein, a novel partner, interacts with DEV UL24 protein
Source: Virol J. 2017 Aug 29;14:166. doi: 10.1186/s12985-017-0830-5 (PMC5575879; doi:10.1186/s12985-017-0830-5)

**A**

**pCMV-Myc  
-UL24**

**Marker**

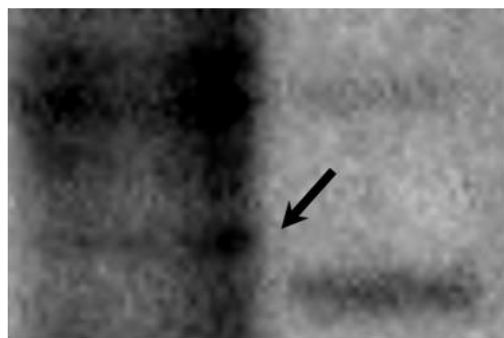

— 66.2KD

— 45KD

**B**

**Marker pCMV-Flag  
-UL54**

60KD —

40KD —

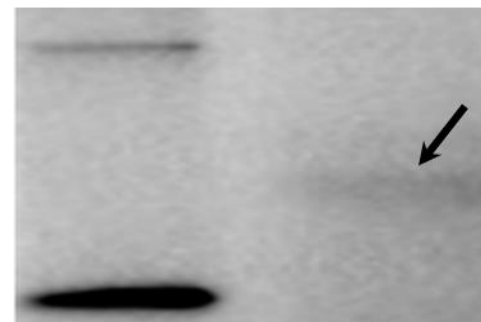

Supplement: Supplementary file 2 — WB analyzed the expression of UL24-fusion protein and UL54-fusion protein in HEK293T cells. HEK293T cells were transfected with eukaryotic plasmid pCMV-myc, pCMV-myc-UL24, and pCMV-Flag-UL54 respectively. At 48 h post-infection, the 293 T cell extracts were carried out Western blotting analysis, which indicated that myc-UL24 and Flag-UL54 was expressed in 293 T cells and the molecular mass of fusion protein is about 45 KD, 50.5 KD respectively. Primary Abs against myc-UL24 and Flag-UL54 were serums of rabbit against UL24 and mouse against Flag respectively. (PDF 44 kb) [file 12985_2017_830_MOESM2_ESM.pdf]
